# Notes on the Nest Architecture and Colony Composition in Winter of the Yellow-Legged Asian Hornet, *Vespa velutina* Lepeletier 1836 (Hym.: Vespidae), in Its Introduced Habitat in Galicia (NW Spain)

**DOI:** 10.3390/insects10080237

**Published:** 2019-08-02

**Authors:** Xesús Feás Sánchez, Rebecca Jane Charles

**Affiliations:** 1 Academy of Veterinary Sciences of Galicia, Edificio EGAP, Rúa Madrid, No. 2-4, 15707 Santiago de Compostela, (A Coruña), Spain; 2 The Larches, Unit 71, Nuneaton CV10 0RQ, UK

**Keywords:** Asian hornet, *Vespa velutina*, nest, colony, winter, Galicia, invasive species

## Abstract

Fifteen years ago, at least one multimated female yellow-legged Asian hornet (*Vespa velutina* Lepeletier 1836) arrived in France, giving rise to a pan-European invasion, altering the environment, affecting ecosystem processes, and impacting society. During winter, *V. velutina* nests (n = 3) were collected in Galicia and data on internal and external aspects of the nests and the colony as a whole were collected. The whole colony population (WCP_N_; adult insects, larvae, and pupae in percentages) was as follows: nest A: n = 176 (49%, 3%, and 48%); nest B: n = 1979 (52%, 36%, and 12%); and nest C: n = 662 (5%, 27%, and 8%). The adult insect population (IAP_N_; males, workers, and gynes in percentages) was as follows: nest A: n = 87 (11%, 66%, and 23%); nest B: n = 1021 (3%, 62%, and 35%); and nest C: n = 430 (20%, 73%, and 7%). As a small number of queens is sufficient for a population to develop, it is necessary to avoid continued spread by deactivating and removing all nests, even those detected in winter. This practice can be of greatest importance in border areas where *V. velutina* is expanding into new territory.

## 1. Introduction

Galicia, located on the western end of the Iberian Peninsula (Europe), is a considerable distance for an insect to travel from Asia, the Americas, or Africa. Despite this, a number of species have migrated in the past 15 years and successfully colonized and spread, resulting in a broad range of consequences to recipient ecosystems and, thereby, human society [1]:

- The Asian chestnut wasp (*Dryocosmus kuriphilus* Yasumatsu 1951 (Hymenoptera: Cynipidae)) was detected in 2004. It has caused significant losses in chestnut production and weakened vitally important chestnut agroforestry cultivars.
- The potato flea (*Epitrix similaris* Gentner 1944 (Coleoptera: Chrysomelidae)) and the Guatemalan moth (*Tecia solanivora* Povolny 1973 (Lepidoptera: Gelechiidae)) were detected in 2009 and 2015, respectively. Their presence has important implications, and the growth of the potato is now forbidden in some areas—a plan implemented by an invasive species task force.
- The red palm weevil (*Rhynchophorus ferrugineus* Olivier, 1790 (Coleoptera: Curculionidae)) was detected in 2013. The species actively destroys the interior of the palm tree.
- The Asian boxy moth (*Cydalima perspectalis* Walker, 1859 (Lepidoptera: Crambidae)) was detected in 2014. Its larvae defoliate the boxy bush.
- The African citrus psyllid (*Trioza erythreae* Del Guercio, 1918 (Hemiptera: Triozidae)) was detected in 2014. It weakens trees, thereby deforming them, and is a vector for various diseases.
- The yellow-legged Asian hornet (*Vespa velutina* Lepeletier 1836 (Hymenoptera: Vespidae)) was detected in Galicia in 2012.

*V. velutina* is naturally distributed in Southeast Asia, India, and China. It was first detected outside of its native habitat in South Korea in 2003 [2] and France in 2004 [3]. The species has subsequently expanded its range and is now present on islands of Japan as of 2012 [4] and on the Japanese western mainland since 2015 [5]. It was soon recognized as a pan-European threat after being detected in Spain (2010) [6], Portugal (2011) [7], Belgium (2011) [8], Italy (2012) [9], Germany (2014) [10], and the Netherlands (2018) [11], as well as on islands such as Majorca in the Balearic Islands (2015) [12], England [13], and the Channel Islands (2016) [14].

Asian hornets are primitively eusocial insects with a colony-based annual life cycle and a caste-based social system, with queens, workers, and males reaching peak abundance at different stages in the cycle. The colonies are founded by a single overwintered inseminated female (haplometrosis). The foundress *V. velutina* queen begins to build the nest, which is the physical structure that will house the incipient colony. Like those of many other social insects, *V. velutina* colonies are usually considered to be mobile entities, the nests shifting from one location to another at some stage in the life of the colony [15]. Nest construction and architecture are the result of a trade-off between thermoregulation and hunting for food, as these are believed to be the most important elements for securing the survival and maximizing the number of offspring in the colony. Maximizing offspring is a major priority for social insect colonies; indeed, it is the driving force behind their evolutionary development and adaptation [16]. Social insect nest structures can vary notably in terms of shape and materials used, but the common objective of the different designs found in nature is the preservation of a stable, greater-than-ambient brood nest temperature. A few structural features are critical for maintaining heat, while others are effective at dissipating it [17]. Studies have proved that temperature regulation in social insect nests can be remarkably precise and may contain ramifications for the coordination of heating and cooling mechanisms without the benefit of an individual thermodirector [18].

**Figure 1:**
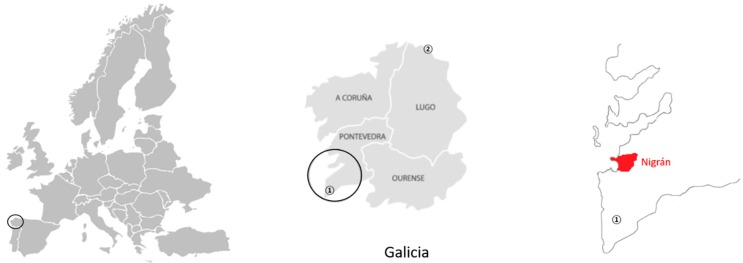
The left image shows the location of Galicia in Europe. The center image shows a map of Galicia marking the two districts where *V. velutina* was first detected in 2012: (1) O Rosal and (2) Burela. The right image shows the sampling region (Nigrán).

The spread of *V. velutina* throughout Galicia has been very rapid. The species was first detected in 2012 in two areas: one nest in Burela (province of Lugo) and one nest in O Rosal (province of Pontevedra). Both nests were destroyed. The distance between Burela and O Rosal is 226 km (see map in Figure 1). The following year, a total of 17 *V. velutina* nests were destroyed. The total number of *V. velutina* nests that were found and eliminated increased from 2 to 10,642 in just four years [19]. However, these data do not give the full extent of the expansion of the *V. velutina*, since many nests destroyed by beekeepers and private enterprises were not reported.

Historically, beekeepers have been the “eyes and ears” in the early detection of this invasive species, given that the diet of these hornet colonies is based on bees and other insects. Although the impact they may have on wild insect populations is still unknown, the species has become a major threat to beekeeping and pollination. Pollination is a fundamental process for the maintenance of life on earth and bees are the main and most effective pollinators [19]. Beekeeping provides an important service to ecosystems, contributing to the improvement of biodiversity while helping to maintain ecological balance.

*V. velutina* is not only an issue for beekeepers. Other productive sectors (forestry, fruit growing, and viticulture) as well as human health and social activities in rural areas and cities have been affected to a greater or lesser extent [20]. The magnitude of the *V. velutina* invasion is reflected by the large number of calls received by emergency services “112 Galicia” related to *V. velutina*. In 2016, emergency services received 13,054 calls due to *V. velutina*, representing a 183% increase from 2015. Emergency calls related to *V. velutina* incidents are one of the highest reasons for calling emergency services in Galicia.

Accidents are common and reported in the local press. Although they caused three deaths in Galicia in 2018 and one death in Nigrán in 2019, there is a general lack of data regarding the health risks of *V. velutina* stings. Poison Control Centers in France have noted that the increase in the Asian hornet population in the southwestern region of the country has not thus far resulted in an increase in the number of Hymenoptera stings [21]. In China, however, the Asian hornet is considered to be a dangerous predatory species. During the summer of 2013, hornet attacks were the cause of 42 human deaths and 1675 injuries in three cities in Shanxi Province. In-hospital mortality of 1091 sting victims was 5.1% from 2009 to 2011 in Hubei Province, a surprisingly high mortality rate compared to other poisonous animals in a single region [22]. In South Korea from 2010 to 2014, there were 483,233 calls requesting removal of wasp nests and Hymenoptera stings caused 78,860 injuries and 49 deaths, with *Polistes rothneyi koreanus Vecht* and *V. velutina* the most prevalent sources. Total medical costs associated with wasp stings over a five-year period were approximately US$3.2 million [23]. All this information could signal that *V. velutina* will be a major problem in cities in the future.

The patterns, processes, and impacts of urban invasions differ significantly from invasions in other environments, posing unique and increasingly complex challenges [24]. Preliminary data on *V*. *velutina* management demonstrate that it has settled in Galician cities. In 2015, three *V. velutina* nests were destroyed in Santiago de Compostela, the capital of Galicia, which has an area of 220 km^2^ and a population of 95,966. By the end of 2017, a total of 971 nests had been destroyed. Handling and destruction of *V. velutina* nests accounted for 52.47% of total calls to the city Corps of Firefighters, more than for any other issue [20]. Major cities would benefit from preparation of an action plan. Several strategies to manage *V. velutina* vary in their effectiveness and there has been little success to date to restrict its invasion and economic, ecological, and social impacts [25]. Methods for detecting and destroying *V. velutina* nests have to be prioritized in terms of performance [25,26].

Many biological factors critical in assessing management strategies for *V. velutina* are still unknown [27], specifically, more knowledge about the ecology, phenology, behavior, and life history. The goal of this study was to describe the nest architecture and colony composition of *V. velutina* nests in winter, the first such study conducted during January within its introduced or native range.

## 2. Materials and Methods

### 2.1. Sampling Area

Three nests were removed on 2, 5, and 11 January 2019. All nests were located in the municipality of Nigrán (Galicia, Spain) and were labeled and identified as A (42°06′54.8″ N, 47′02.6″ W), B (42°06′47.0″ N, 8°47′49.7″ W), and C (42°08′48.2″ N, 8°48′37.7″ W), respectively (Figure 1). Nigrán is a 35 km^2^ municipality and contains the metropolitan area of Vigo, the most populous city in Galicia with a population of approximately 18,000. It is surrounded to the west by the Vigo estuary and the Atlantic Ocean and has a temperate climate and a greatly varying orography, including wide valleys. The highest altitude is approximately 500 m. The coast is composed of beaches and mountainous forests. Two rivers flow into the coast of Nigrán: the Miñor and the Muiños, creating an estuary that turns into marsh.

### 2.2. Nest Collection

**Figure 2:**
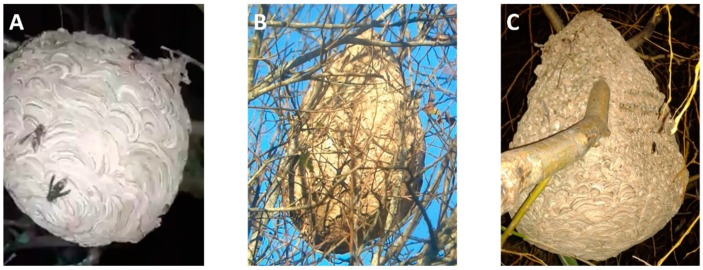
The *Vespa velutina* nests identified as (**A**–**C**) prior to their removal on 2, 5, and 11 January of 2019, respectively.

All of the nests (Figure 2) were detached and removed during the night and a quick-drying foam was used to seal the nest entrance. Smaller branches of the tree that had become part of the nest were removed and height of the nest (H_N_) from the ground was recorded. The nests were sealed using two bags, 105 × 85 cm in size, and the main branch of the tree to which the nest was secured was manually cut using a hand saw. The nests were placed in a freezer for 12 h at a temperature of −18 °C.

### 2.3. Nest Structural Features

Various external and internal measurements were taken of nests A–C to describe their form and structure. The measurements were carried out using a Vernier caliper, a ruler, and measuring tape.

#### 2.3.1. External

The overall length (L_N_), width (W_N_), and circumference (C_N_) of the nests were recorded. The volume of the nest (V_N_) was calculated using the formula of a prolate spheroid:

V_N_ = 4/3 π × (L_N_/2)² × (W_N_/2) (1)

Tree branches inside the nest were denoted as main (M_B_) and secondary (S_B_) branches. The number of secondary branches (N_SB_), length of the main branch covered by the nest (L_MBC_), and the diameter of both main and secondary branches (D_MB_ and D_SB_) were recorded.

#### 2.3.2. Internal

After external measurements and inspection, the nests were dissected. The number of combs (N_C_), with their maximum (L_C-max_) and minor (L_C-min_) lengths, were recorded and the number of pillars (N_P_) that connected the combs was recorded. The presence of upholstered cells (UHCs) was recorded in each comb from all nests. Surface of the combs of the nest (TS_N_) and the surface of the combs (S_C_) were calculated using the formula for the area of an ellipsoid:

S_C_ = π × (L_C-max_/2) × (L_C-min_/2) (2)

The total cells (TC_C_) of a given comb and the total cells of a nest (TC_N_) as ∑TC_C_ were calculated using the following formula:

TC_C_ = ((3n/2) + 1) × n/2 (3)

where “n” is the number of cells counted along its longest diameter [25].

### 2.4. Nest Colony Composition

The number of adult individuals was counted manually, obtaining the total insect adult population of each nest (IAP_N_). Females were differentiated from males by the presence of 12 segmented antennae (males have 13). Males have an apex of the last sternite bilobate which is sharp in females. The difference between workers and gynes was established at a mesoscutum width of 4.5 mm [28].

The whole colony population of each nest (WCP_N_), that is, adult insects and nonmature (larvae and pupae), present in different combs of the nests was recorded. Later, IAP_N_ were separated into males, workers, and gynes.

The theoretical adult production (TAP_N_) of individuals of each nest was inferred using the following function or method function [26]:

TAP_N_ = 7.12 × L_C-max_^2^ − 37.72 × LC-max − 9.68 (4)

where TAP_N_ is the number of individuals produced by the colony and L_C-max_ is the diameter of the largest comb.

## 3. Results

### 3.1. External Nest Structure

All nests were suspended from trees at heights of 11, 12, and 10 m for nests A–C, respectively. Nests A–C were found in tree species *Alnus glutinosa*, *Salix alba*, and *Salix babylonica*, respectively. In all nests, a branch of the tree was identified as being inside the nest, covered by it to varying lengths and serving as the main nest support. Nests B and C had additional secondary branches, six and four, within the top structure. The length (L_N_), circumference (C_N_), width (W_N_), volume (V_N_), and height (H_N_) from the ground of all nests are shown in Table 1. Nest A was the smallest and had a spheroidal shape with a volume of 9.7. Nests B and C had more of a pear shape, with calculated volumes of 101.8 and 57.0, respectively.

A detailed inspection of nest A revealed it was completely covered with various fragile leaves. Nest A was generally brown in color with strips of alternating lighter and darker shades, indicating that various sources of material were used in its construction. Nests B and C were externally covered mostly by spit pulp and only sheets of paper covering the bottom of these nests were found. The outsides of nests B and C were more durable than nest A, as they did not crumble with pressure upon handling. Nests B and C were externally a bright and homogenous tone. Nest C externally had a slight “varnish” or shell-like effect and possessed a distinct beehive aroma. It appeared nest A was formed using large sheets of paper, yet nests B and C only had this laminar structure in the lower part of the nest, the remaining parts externally covered with sculpted or stuck pulp on the surface.

### 3.2. Internal Nest Structure

Nest A was soft with a friable envelope consisting of different numbers of sheets (between 6 and 10) overlapping, covering the inner chamber formed by the combs. Nests B and C possessed a rigid structure with hard and fibrous envelopes. Only in the lower portion was it possible to manually remove the sheets (between four and six) wrapping this part of nests B and C.

**Figure 3:**
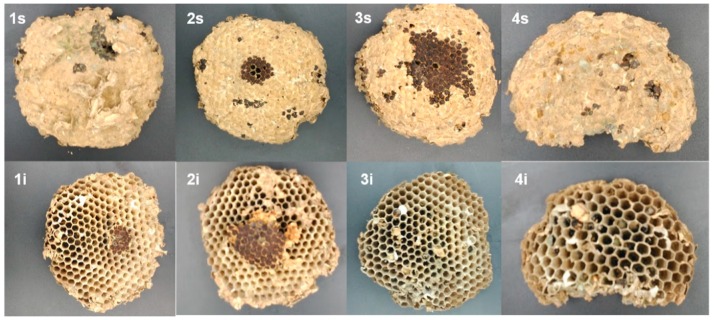
Combs taken from *V. velutina* nest A. The figures show both superior (s) and inferior (i) views of the combs labeled from top to bottom (1–4) in the same order that they were found within the nest.

**Figure 4:**
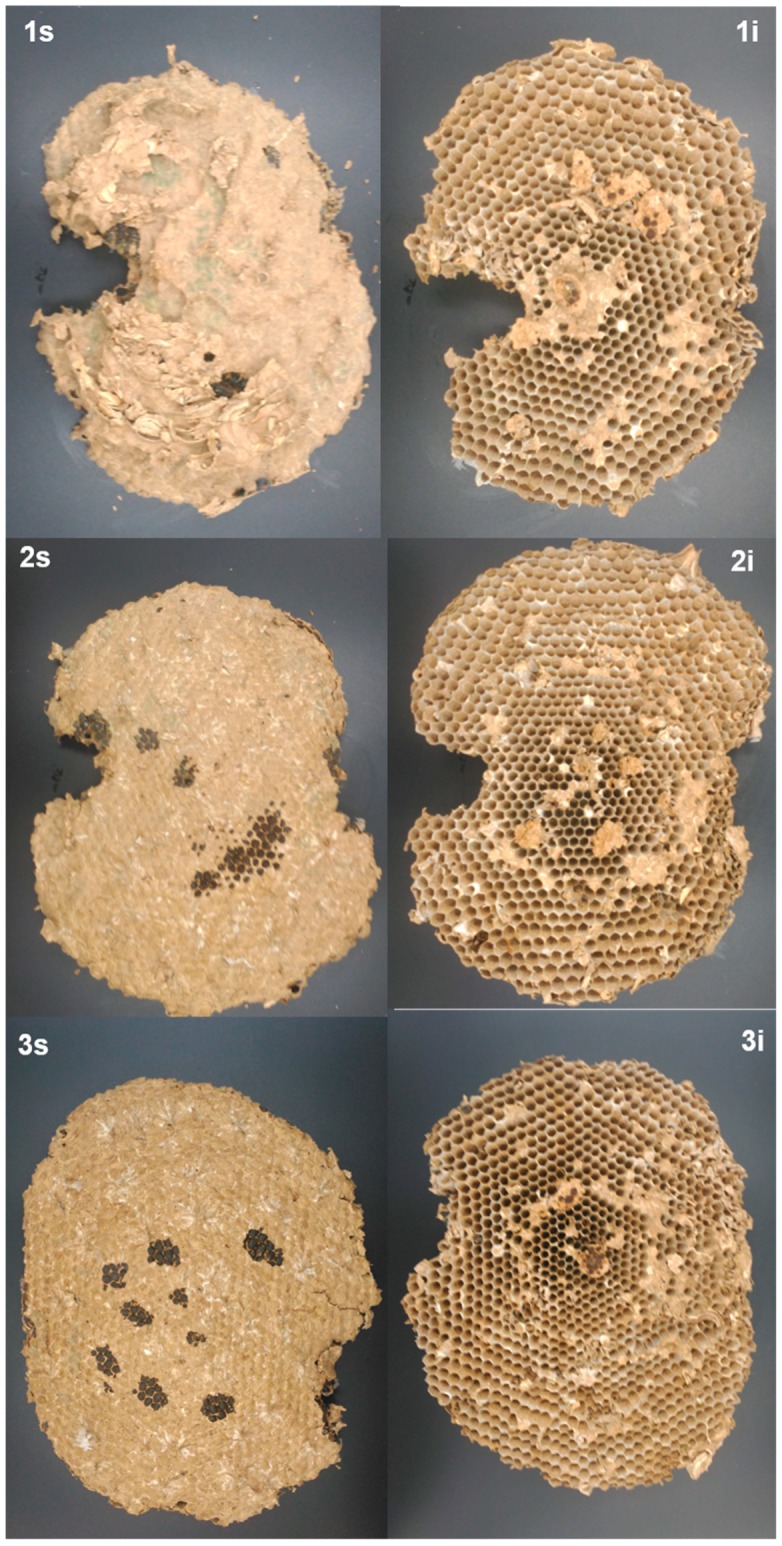

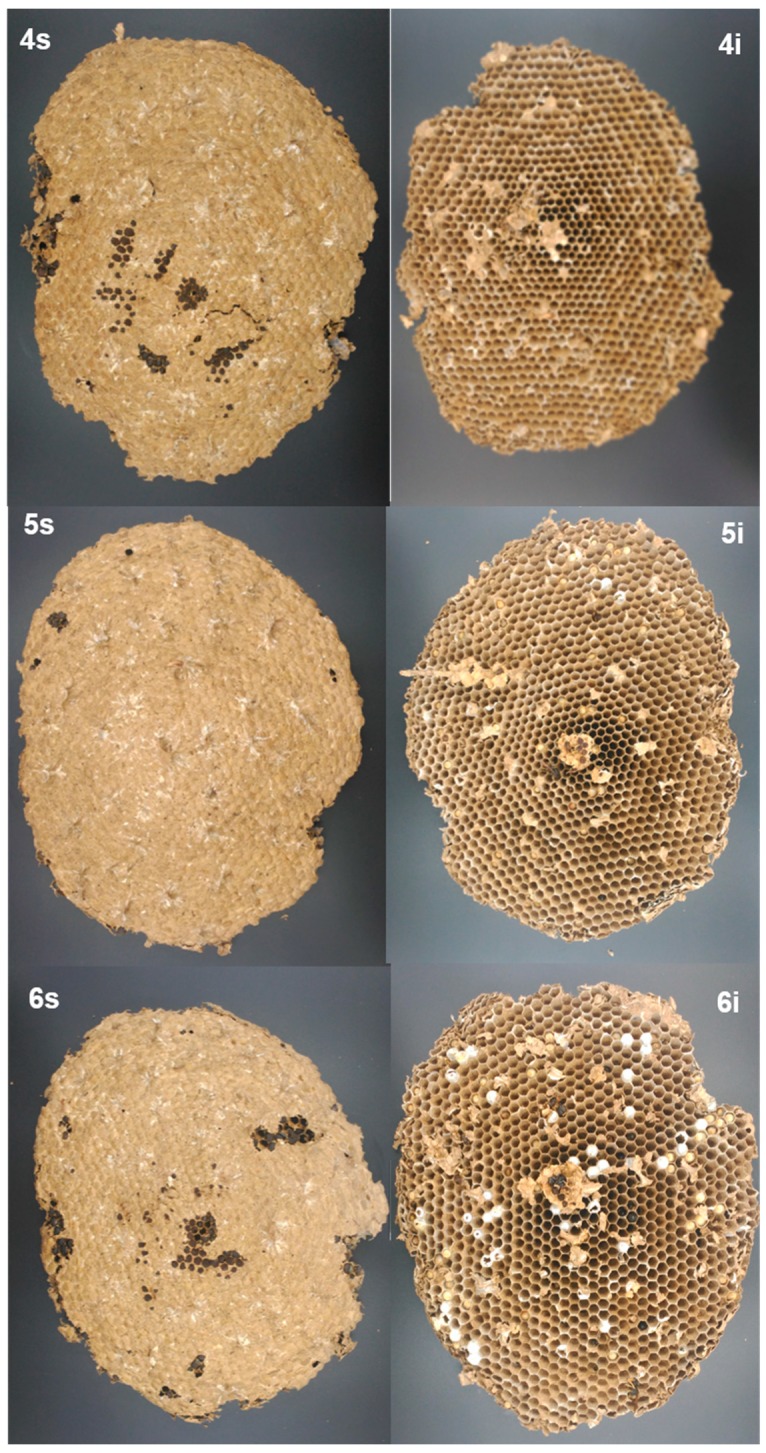

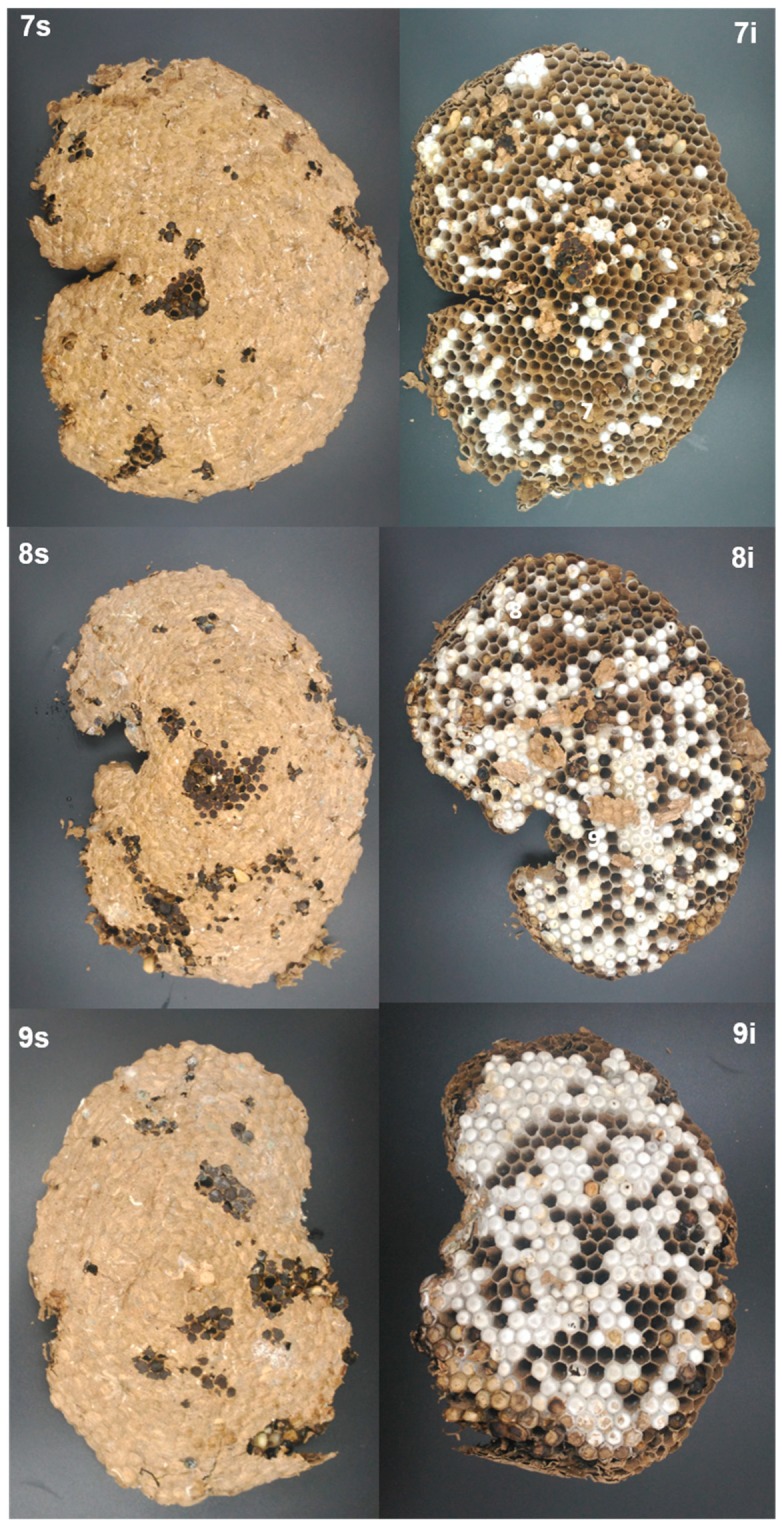
Combs taken from *V. velutina* nest B. The figures show both superior (s) and inferior (i) views of the combs (n = 9) labeled from top to bottom (1–9) in the same order that they were found within the nest.

**Figure 5:**
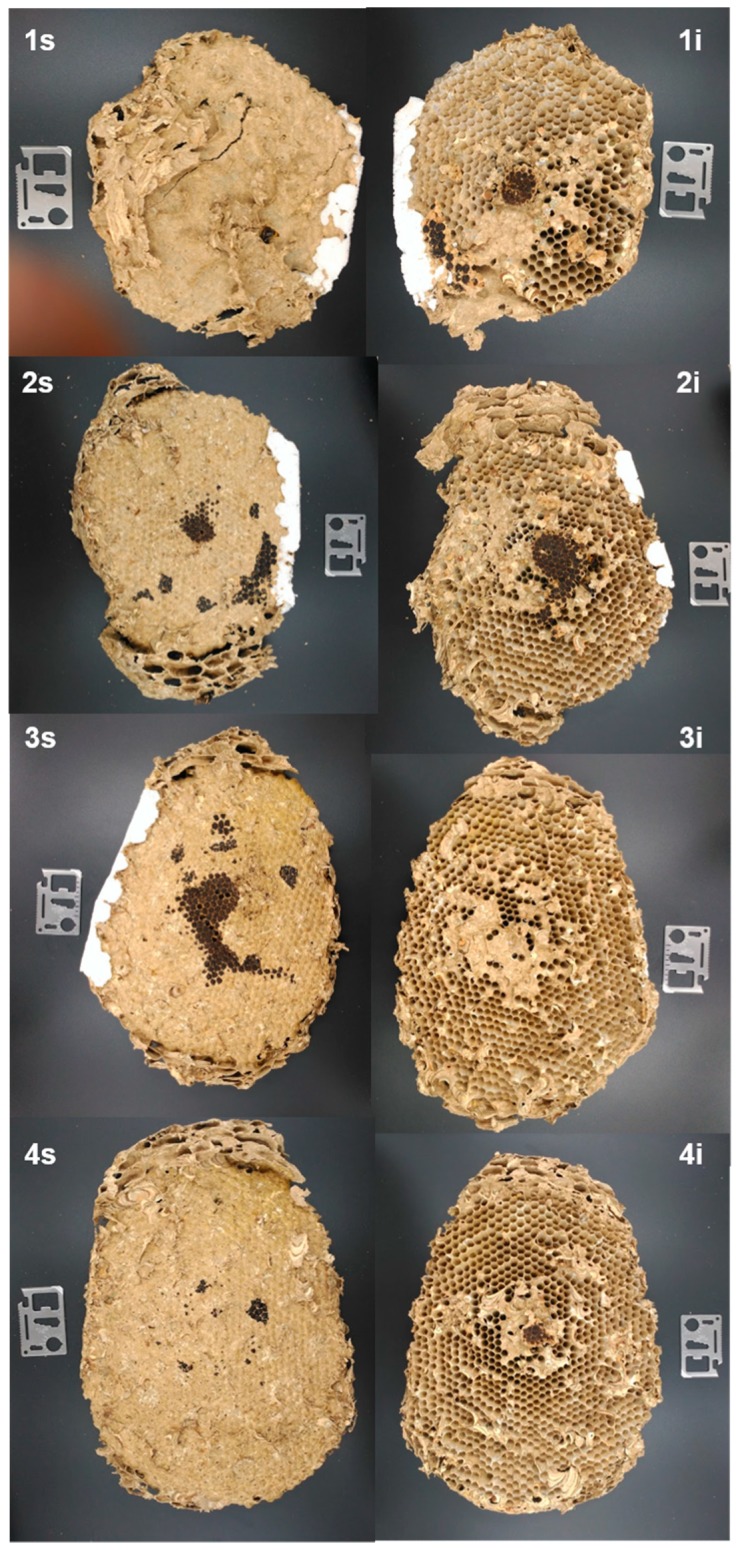

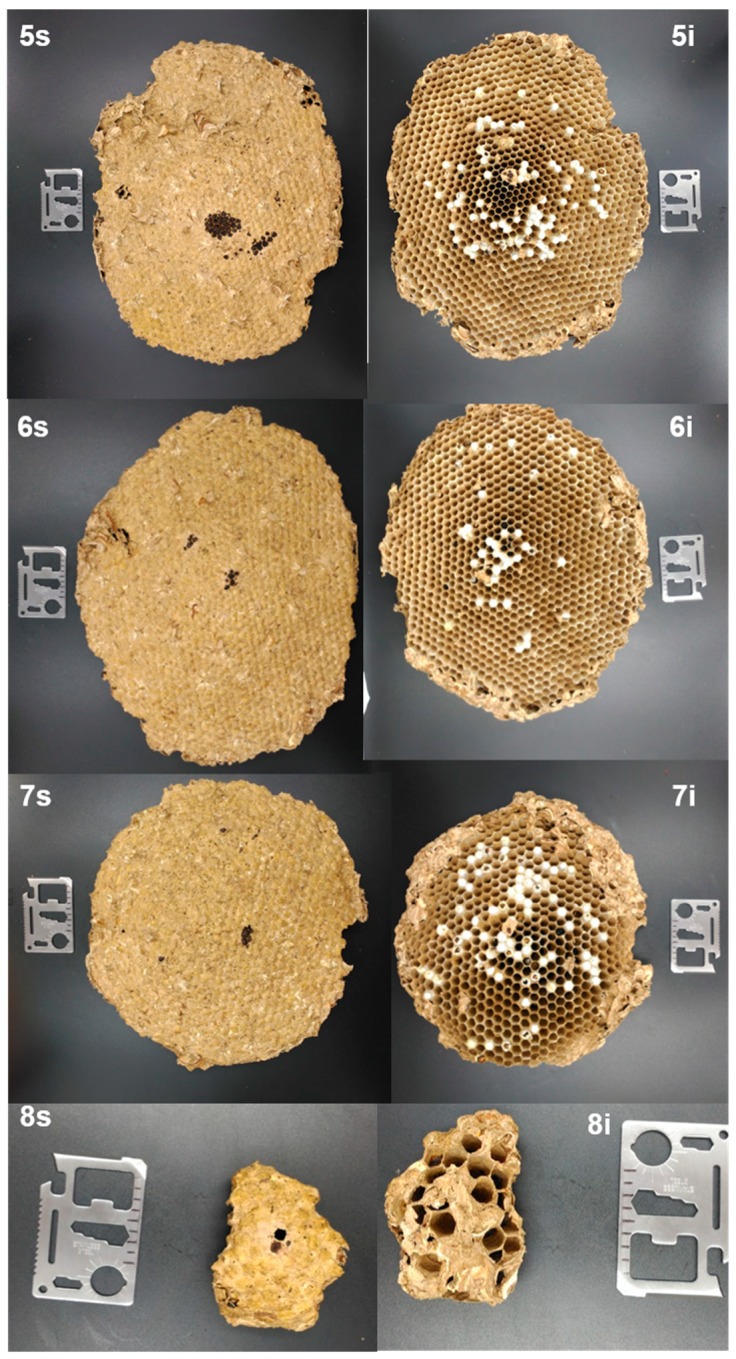
Combs taken from *V. velutina* nest C. The figures show both superior (s) and inferior (i) views of the combs (n = 8) labeled from top to bottom (1–8) in the same order they were found within the nest. The metal piece shown in each figure is 6 cm in length.

Once the outer cover was removed from all nests, mostly found were (a) a conical top or roof, with lengths of 8, 25, and 21 cm for nests A–C, respectively, and (b) the sets of the combs that made up the internal chamber, with four, eight, and nine combs found in nests A (Figure 3), B (Figure 4), and C (Figure 5), respectively.

In nest A (Figure 3, 1s), it was noted that the first comb was directly joined to the support branch of the tree, having a smaller roof cone surrounding it. In the other nests (B and C), the first combs did not attach directly to the branch (Figure 4, 1s and Figure 5, 1s).

There was an absence of UHCs in all combs of nest A, but UHCs were present in nest B (combs 1–6) and nest C (combs 1–5 and 8).

There was a free internal space that laterally separated the internal part of the outer cover of the nest and the internal chamber of the combs, between 3 and 5 cm. However, in nest C, this space was not always present, establishing several connections between the inner wall of the outer cover and the outer edges of the combs (Figure 5, combs 2–4).

**Figure 6:**
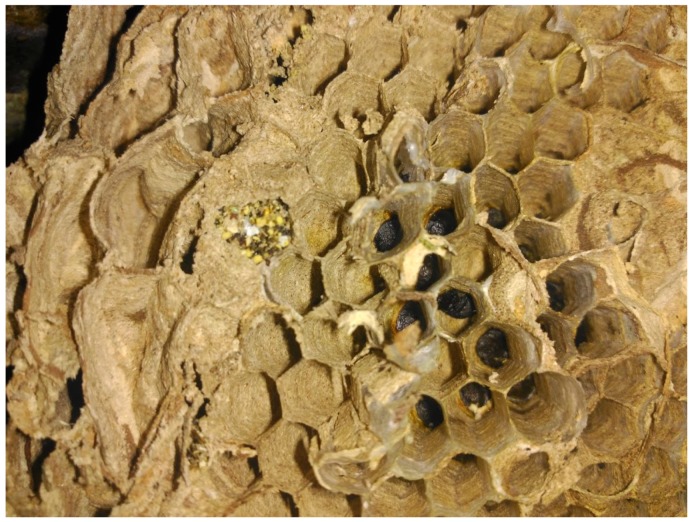
Nest C shows a yellow pollen-like material found inside a pocket.

Inside nest C (Figure 6), we found pollen loads in a pocket of the nest envelope. Unfortunately, we could not conclude if this was accidentally included in the colony due to tree growth and packing by insects or if it was intentionally recovered and stored by the hornets.

Total surface of the combs for each nest, in decreasing order, was nest B > nest C > nest A. Nest B contained the largest number of combs (nine combs), followed by nest C (eight combs), and A (four combs). In nest C, comb 5 had the greatest length (L_C-max_ = 36.1 cm) of all combs of the three nests studied (Table 2).

The theoretical adult production (TAP_N_) was 1129 for nest A, 7492 for nest B, and 7926 for nest C. The total cells of the nest (TC_N_) was 1074, 9962, and 7784 for nests A–C, respectively.

### 3.3. Nest Colony Composition

All nests contained adult insects, eggs, larvae, and pupae with varying distributions in the combs (Table 3). Whole colony populations (WCP_N_) were 176, 1979, and 662 for nests A–C, respectively. The IAP_N_ showed the same tendency, with nest A as the most populated with 1021 individuals, followed by nests C and B. However, in nest A, more larvae were found (n = 85) than in nest C (n = 50). In upper combs, there were no larvae, pupae, or eggs. Immature stages were found in lower combs, concretely in combs 3 and 4 in nest A, combs 5–9 in nest B, and combs 5–7 in nest C (Table 3).

**Figure 7:**
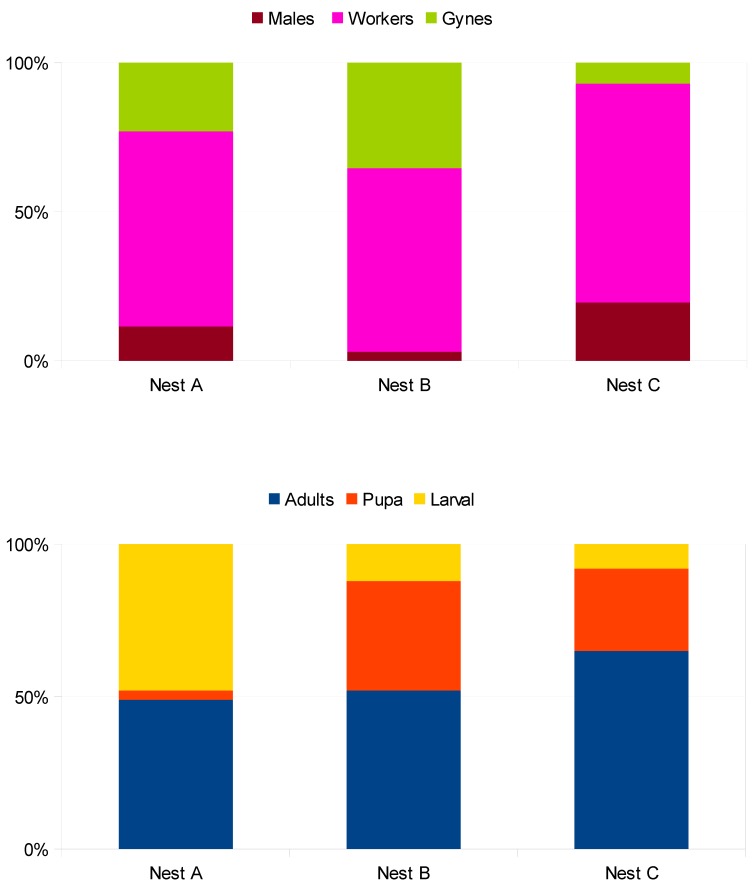
Percentage of the whole colony population (adults, larvae, and pupae) and insect adult population (males, workers, and gynes) of the *V. velutina* nests studied.

In each nest, the three castes—males, workers, and gynes—were found in different percentages (Figure 7). The WCP_N_ of adults, larvae, and pupae were as follows: Nest A (n = 176) with (49%, 3%, and 48%), Nest B (n = 1979) with (52%, 36%, and 12%), and nest C (n = 662) with (65%, 27%, and 8%). The IAP_N_ of males, workers, and gynes were as follows: Nest A (n = 87) with (11%, 66%, and 23%), Nest B (n = 1021) with (3%, 62%, and 35%), and Nest C (n = 430) with (20%, 73%, and 7%).

## 4. Discussion

Approaching active colonies of Vespidae in general, and *V. velutina* in particular, is complicated and potentially risky. Vibrations, impacts, or panicked movements near a colony may be perceived as threats, provoking a defensive attack. However, *V. velutina* colonies reportedly show unsolicited assault behavior and the recommended minimum distance to observe a colony is several meters [29,30]. The observation of Vespidae nest structure is quite difficult, as one cannot generally study a given nest thoroughly using nondestructive methods. Details regarding seasonal modifications need to be obtained by breaking open nests [26].

Study nests were collected in Nigrán, 25 km away from O Rosal, one of the first two original entry points of *V. velutina* detected in Galicia in 2012. The nests represented the seventh annual known generation of *V. velutina* in Galicia. The collection protocol ensured complete sampling of the whole colony without using pesticides or otherwise damaging the nest. In previous studies, nests were obtained during the day; however, as *V. velutina* does not possess nocturnal habits, such as the European *Vespa crabro*, this enabled nighttime collection to include all adult individuals present.

Study nests were located in trees at heights greater than 10 m. *V. velutina* is a well-known aerial-nesting species within and outside its native range but also displays a broad and flexible nest site preference, introducing difficulty in its detection and destruction. They can build nests in trees at high altitudes (between 10 and 40 m) in trees in urban areas and also at lower altitudes in fruit trees, shrubs, and bushes in cities, rural areas, and gardens. It takes advantage of human construction to settle at high altitudes in buildings, on electricity pylons, houses, and lamp posts or at lower heights (less than 5 m) in construction interiors (attics and ceilings of barns and warehouses) [29].

A geo-referenced map of *V. velutina* nests in France revealed that of 6073 nests, 48.5% were observed in artificial and 42.25% in agricultural surroundings. Less than 10% occurred in natural areas (8.1%) and wetlands (1.1%). The majority (70.0%) were located more than 10 m above the ground, 26.3% were between 2 and 10 m above the ground, and only 3.7% were situated <2 m above the ground. In addition, 87.0% of the 3296 nests were built in vegetation (trees, shrubs, bushes, etc.), 12.8% on construction (buildings and/or houses), and only 0.2% underground [27]. This demonstrates that *V. velutina*, like *Vespa simillina*, can nest in a diverse range of places with considerable adaptability, and both species are able to change nest sites in response to their environment [31]. Nests at high altitudes in trees can be dangerous to forestry management (wood cutting) personnel working in cities or forests. In 2018, many accidents were incurred by forestry workers due to the presence of *V. velutina* undetected in tree canopies, which required the mobilization of aerial transport to transfer the injured to the nearest health center.

The nest envelope of *V. velutina* has been characterized as a shell-like structure with short air pockets created using a maximum of five layers [10]. A nest may be defined as any modification of the environment by adult insects that provide shelter for the rearing of their offspring [32]. *V. velutina* make their nest out of paper-like fiber. Wood pulp collected from different sources is chewed and mixed with saliva by the hornets. The size of a hornet nest grows in proportion to the size of the inhabitants and the development rate of the colony. In our study, the insect adult populations (males, workers, and gynes) were as follows: nest A, n = 87 (11%, 66%, and 23%); nest B, n = 1021, (3%, 62%, and 35%); and nest C, n = 430, (20%, 73%, and 7%). Previous work showed that the number of combs, cells, and individuals produced from nests (n = 3) in December in France varied between 7 and 10 (mean of 8), 3685 and 5607 (mean of 4756.7), and 4039 and 4583 (mean 4382.4), respectively [26]. A nest removed in Germany in November consisted of a fully developed, medium-sized nest of six honeycombs with approximately 2637 used brood cells.

Availability of food and nesting resources is important to insects, as well as weather and climatic variables, as the thermoregulation capability of the nest is determined by ambient temperature. Nest architecture is an extended phenotype of a colony and thus under natural selection. Within a species, the rule may be context dependent, varying adaptively according to colony age, size, nutritional status, or reproductive state [33]. Previous research suggests that seafood might provide a valuable alternative food source favoring *V. velutina* colony development [34]. In the area from which nests were collected in the present study, *V. velutina* has a long colony cycle; the queens are normally spotted awakening from hibernation as early as late February. The present work shows colonies are active in January.

Different organizations, mainly public administration and academia, share the opinion that “the destruction of nests in winter is not seen to be an important method of control since the queen is dead and the future founders have already left the nest to winter” [35,36]. However, the results obtained in the present work show us that three nests captured in winter were active with a variable number of individuals, among which were a non-negligible number of founding queens. Since only one or a small number of queens is sufficient for the development of the population, it becomes necessary to change previous approaches to avoid future spread. Any detected nest must be immediately deactivated and removed. This may be especially important in areas where *V. velutina* is expanding into new territories.

An in-depth understanding of the behavioral ecology of invasive species in unique surroundings is needed to determine the behavior that maximizes health and thereby enlarges the alien species population [37,38]. A *V. velutina* colony life history can be described by understanding age and size at maturity, pattern of reproductive periods, size and number of offspring produced, and demise of the colony [39]. Better knowledge of invasive species is important to address the most suitable actions to prevent their spread. Several important questions we could not address include whether the queens found in the nests belonged to the nests originally, if they were fertilized or not, their age, or if they were using the nest as protected space while waiting for better conditions to start new nests and colonies or as a stop prior to hibernation.

## 5. Conclusions

To the best of our knowledge, the work presented here represents the first reported study of nest architecture and colony composition over winter of the yellow-legged Asian hornet. Since all overwinter nests contained living individuals of all castes, as well as brood and eggs, it is extremely important to remove *V. velutina* nests during the winter as well as the rest of the year. It is essential to work together to try to reduce or stop the spread of this invasive species by way of educating the public about *V. velutina* and supporting further research with a greater number of nest studies in different areas and at different times of their development.

## Figures and Tables

**Table 1:** External nest structural features. Nest length (L_N_), nest circumference (C_N_), and nest width (W_N_) in centimeters. Volume of the nest (V_N_) in liters. The height of the nest (H_N_) from the ground (in meters). The number of secondary branches (N_SB_), length of the main branch covered by the nest (L_MBC_), and the diameter of both the main and secondary branches (D_MB_ and D_SB_) in centimeters.

Nest	L_N_	C_N_	W_N_	H_N_	V_N_	Tree	L_MBC_	D_MB_	N_SB_	D_SB_
A	28.7	69.3	22	11	9.7	*Alnus glutinosa*	17	1.9	-	-
B	71.6	119.2	37.9	12	101.8	*Salix alba*	34	2.7	6	0.6; 0.9; 1; 1.4 and 1.5
C	54.4	115.6	36.8	10	57.0	*Salix babylonica*	2.5	3.7	4	1.4; 1.6; 2.5 and 2.6

**Table 2:** Internal nest structural features. Number of the combs (N_C_), with their maximum (L_C-max_) and minor (L_C-min_) length, and number of pillars (N_P_). Surface of the combs (S_C_). Total surface of the combs (TS_C_). Total cells (TC_N_) of *V. velutina* nests studied.

	Nest A				Nest B				Nest C		
Comb num.	L_C-max_	L_C-min_	S_C-max_	N_P_	L_C-max_	L_C-min_	S_C-max_	N_P_	L_C-max_	L_C-min_	S_C-max_
1	14.2	13.1	146.1	12	27.1	21.9	466.1	8	25.1	24.2	477.1
2	14.0	14.0	153.9	13	31.2	26.1	639.6	30	28.9	22.1	501.6
3	16.1	15.6	197.3	12	31.3	27.2	668.7	47	32.1	24.3	612.6
4	13.3	12.2	127.4	15	32.9	30.9	803.6	43	34.5	24.2	655.7
5	-	-	-		35.2	30.3	837.7	46	36.1	26.2	742.8
6	-	-	-		33.1	29.2	759.1	43	33.9	25.8	686.9
7	-	-	-		31.0	25.2	613.6	42	24.9	22.9	447.8
8	-	-	-		29.1	21.2	484.5	22	7.9	6.1	37.8
9	-	-	-		24.2	19.3	366.8	14	-	-	-
V_min_	13.3	12.2	127.4	12	24.2	19.3	366.8	8	7.9	6.1	37.8
V_max_	16.1	15.6	197.3	15	35.2	30.9	837.7	47	36.1	26.2	742.8
N_C_	4				9				8		
TS_N_	624.7				5639.6				4162.5		

V_min_ = minimum value; V_max_ = maximum value.

**Table 3:** Nest colony composition of the *V. velutina* nests studied. Insect adult population (IAP_N_) and whole colony population (WCP_N_).

Nest	IAP_N_	WCP_N_	Comb number	Eggs	Larval	Pupa
A	87	176	1	-	-	-
			2	-	-	-
			3	+	22	2
			4	-	62	3
B	1021	1979	1	-	-	-
			2	-	-	-
			3	-	-	-
			4	-	-	-
			5	-	52	2
			6	-	48	42
			7	+	68	143
			8	+	48	285
			9	+	30	240
C	430	662	1	-	-	-
			2	-	-	-
			3	-	-	-
			4	-	-	-
			5	-	12	76
			6	+	11	34
			7	+	27	72
			8	-	-	-
